# Risk Factors Associated with Nursing Home COVID-19 Outbreaks: A Retrospective Cohort Study

**DOI:** 10.3390/ijerph18168434

**Published:** 2021-08-10

**Authors:** Lucia Cazzoletti, Maria Elisabetta Zanolin, Ilaria Tocco Tussardi, Mulubirhan Assefa Alemayohu, Ernesto Zanetel, Donatella Visentin, Luca Fabbri, Massimo Giordani, Giancarlo Ruscitti, Pier Paolo Benetollo, Stefano Tardivo, Emanuele Torri

**Affiliations:** 1 Department of Diagnostics and Public Health, University of Verona, 37134 Verona, Italy; lucia.cazzoletti@univr.it (L.C.); elisabetta.zanolin@univr.it (M.E.Z.); ilaria.toccotussardi@univr.it (I.T.T.); mulubirhanassefa.alemayohu@univr.it (M.A.A.); zanetelernesto@libero.it (E.Z.); donatellavisentin77@gmail.com (D.V.); 2 Azienda Provinciale per i Servizi Sanitari, Autonomous Province of Trento, 38123 Trento, Italy; luca.fabbri@apss.tn.it (L.F.); direzionesanitaria@apss.tn.it (P.P.B.); 3 Unione Provinciale Istituzioni per l’Assistenza, Autonomous Province of Trento, 38122 Trento, Italy; direttore@upipa.tn.it; 4 Department of Health and Social Policies, Autonomous Province of Trento, 38122 Trento, Italy; giancarlo.ruscitti@provincia.tn.it (G.R.); emanuele.torri@provincia.tn.it (E.T.)

**Keywords:** COVID-19, epidemic risk, infection prevention and control, Italy, long-term care, nursing home, outbreak

## Abstract

Background: The coronavirus disease 2019 (COVID-19) pandemic had a devastating impact on nursing homes/long-term care facilities. This study examined the relationship between geography, size, design, organizational characteristics, and implementation of infection prevention and control (IPC) measures and the extent of COVID-19 outbreaks in nursing homes in the Autonomous Province of Trento (Italy) during the time frame of March-May 2020. Methods: The analysis included 57 nursing homes (5145 beds). The association between median cumulative incidence of COVID-19 cases among residents and characteristics of nursing homes was assessed by Mann–Whitney U test, Kruskal–Wallis test or Spearman rho. To evaluate the potential confounding of geographical area, a 2-level random intercept logistic model was fitted, with level 1 units (patients in nursing homes) nested into level 2 units (nursing homes), and “being a COVID-19 case” as the dependent variable. Results: Median cumulative incidence was not significantly associated with any of the variables, except for geographical region (*p* = 0.002). COVID-19 cases clustered in the part of the province bordering the Italian region most affected by the pandemic (Lombardy) (45.2% median cumulative incidence). Conclusions: Structural/organizational factors and standard IPC measures may not predict the epidemiology of COVID-19 outbreaks and be sufficient alone to protect nursing homes against them.

## 1. Introduction

The coronavirus disease 2019 (COVID-19) pandemic, caused by a novel coronavirus (severe acute respiratory syndrome coronavirus 2, SARS-CoV-2), has had a marked impact on every sector of society, with long-term care facilities for the elderly bearing a disproportionate amount of the disease burden and mortality [1].

Long-term care facilities for the elderly encompass, in different countries and healthcare systems, a broad range of institution types, with or without the delivery of skilled nursing care. The COVID-19 pandemic has affected vulnerable people hosted in residential facilities more than any other category, with this group representing between 19% and 72% of all SARS-CoV-2-related deaths [2]. 

Among these facilities, nursing homes has proved to be one of the most at-risk environments for COVID-19 infections globally due to various contributing factors, including the following: the age and co-morbidities of the residents, who are particularly vulnerable to respiratory diseases [3]; the presence of a relatively large number of people congregating together; the nature of the setting, where social activities and community life play an essential role in care processes; the skill mix of the nursing home staff; and as the non-hospital nature of these organizations, characterized by medical technologies and an environment that is often unsuitable for carrying out effective and prolonged patient isolation and clinical treatment. The transmission dynamics of COVID-19, combined with the low availability of testing, have fueled a rapid spread within and between facilities, leading to high morbidity and mortality among residents in these settings [4]. Fatality and morbidity rates were roughly comparable across geographic areas and between continents [5].

Understanding clearly the factors that may have contributed to outbreaks as a result of the pandemic have attracted scientific and legal attention for the purposes of devising policies, action plans and standards to safeguard the safety of both residents and employees in such facilities during the COVID-19 pandemic and beyond [6]. Despite a wealth of individual reports of outbreaks and fatalities in aged care facilities related to COVID-19 [7,8,9,10,11,12], as well as review articles quantifying the outsized impact of this pandemic in the frail populations [3], structural, organizational and practice-related protective factors in these setting have been analyzed to a lesser degree. Several studies, mainly conducted in North America, have investigated the relationship between COVID-19 outbreaks and certain features of long-term care facilities (including nursing homes), such as their geography, size, design, staffing levels, compliance with infection prevention and control (IPC) regulations, health inspections and quality ratings [13,14,15]. However, there is considerable heterogeneity among these studies, in terms of study design (mainly cross-sectional cohort studies), settings, participants, time frame, considered variables, and outcome measures. The limitations that need to be considered when interpreting the results include reliance on cross-sectional data, unmeasured confounding factors (common in observational studies), inaccuracies and inconsistencies in data, and metrics reporting (i.e., cases counts). Further investigations are required to extend such assessments to other contexts, countries and healthcare systems, as well as analyze the effectiveness of specific factors for protecting long-term care facilities against COVID-19 outbreaks. Studying these associations in countries such as Italy, which was hit by the COVID-19 pandemic early and to a significant degree [16,17], with devastating consequences for nursing homes [18], may provide important insights.

The aim of this study was to examine the association between certain measurable factors (structural, organizational and practice-related) and the cumulative incidence of COVID-19 among nursing home residents in the Autonomous Province of Trento, Italy, during the peak of the COVID-19 outbreak, the so-called “first wave” (March–May 2020).

## 2. Materials and Methods

### 2.1. Study Design

This retrospective cohort study of 57 nursing homes in the Autonomous Province of Trento included a total of 5145 licensed beds with full occupancy from 1 March 2020 to 1 June 2020. We analyzed the association between certain characteristics of nursing homes and the risk of COVID-19 cases among residents. The considered factors were the geographic location within the regional territory of the nursing homes, the structural and organizational characteristics, and the implementation of IPC measures. 

### 2.2. Setting

The autonomous province is an alpine territory located in Northeastern Italy, with an area of 6207.12 square kilometers, a population of 545,425 inhabitants, and a life expectancy at birth in 2018 of 82.7 years for males (which is the highest among European regions, whose average is 78.2) and 86.3 for females (versus an European average of 83.7) [19]. Across the region, universal health care is provided by the Italian National Health Service. The number of long-term care beds per 100,000 people aged ≥65 is two times greater than the national average (38.5 in the Autonomous Province of Trento versus 14.6 in Italy) [20]. Nursing home care is delivered by public and private non-profit institutions. Except for very few privately managed beds, all residents are admitted upon a multidimensional need assessment carried out by a multidisciplinary team from the provincial local health authority (Healthcare Trust of the Autonomous Province of Trento), evaluating the degree of non-self-sufficiency and care needs. Although people of all ages may reside in these facilities, the vast majority are elderly.

All of these nursing homes provide their residents with personal care, nursing care (24-h assistance), medical care and rehabilitation, as well as occupational and social activities. Within some nursing homes, there are dementia care units or special care units designed, staffed, and equipped to care for older adults with dementia or other complex clinical conditions requiring more intensive care. 

In this study, we included all nursing homes for the elderly; therefore, we excluded other long-term care settings for people with disabilities (targeting different age groups and needs), as well as hospital-based long-term care.

### 2.3. Outcomes and Data Sources

General data on the nursing homes (size, structure and special care units, urban/rural status, staff, compliance with quality standards, geographic location) were collected from the official sources of the Department of Health and Social Policies of the Autonomous Province of Trento and the Healthcare Trust of the Autonomous Province of Trento and updated as of 1 March 2020.

The Healthcare Trust of the Autonomous Province of Trento, the provincial entity in charge of collecting and reporting all official data since the onset of the pandemic, provided data on cases of SARS-CoV-2 infection detected in nursing home residents and in the surrounding municipality. All diagnoses were confirmed with molecular diagnostics for SARS-CoV-2 (Reverse Transcription Polymerase Chain Reaction, RT-PCR) performed in the provincial reference laboratory. Data were collected between 1 March 2020 and 1 June 2020, corresponding to the entire “first wave” duration in the Autonomous Province of Trento (the first COVID-19 case was detected in the region on March 2 and, at the end of May, new cases were almost down to zero) [21,22,23].

Specific organizational and structural information and data related to IPC measures were retrieved from a study carried out in May 2019 as part of a broad regional project initiated to improve infection IPC programs and tackle antibiotic resistance in nursing homes. The goal was to provide a reliable and detailed baseline of structure and practice-related data on IPC in the real context of nursing homes to leverage the implementation of improvement actions.

In order to collect standardized information on IPC, a questionnaire was designed, piloted, and administered to all nursing homes in the Province. The questionnaire incorporated items from the tool and data collection protocol used in the “Healthcare-Associated Infections in Long Term Care Facilities” (HALT-3, 2016–2017) project, proposed by the European Center for Disease Prevention and Control and validated in the Italian context [24,25], items adapted from the “Infection Prevention and Control Assessment Tool for Long-term Care Facilities” developed by the Centers for Disease Control and Prevention [26], and from another tool tested in an Italian survey related to Legionellosis in Italian healthcare facilities [27]. For the sake of the current study, from the multiple domains and elements investigated in the survey, we extrapolated 13 items related to IPC measures relevant for respiratory infection transmission (see Table 1). The vast majority of nursing homes participated in the survey (45 nursing homes, with a response rate of 79%), guaranteeing a coverage of 75% of the total number of licensed beds.

**Figure 1:**
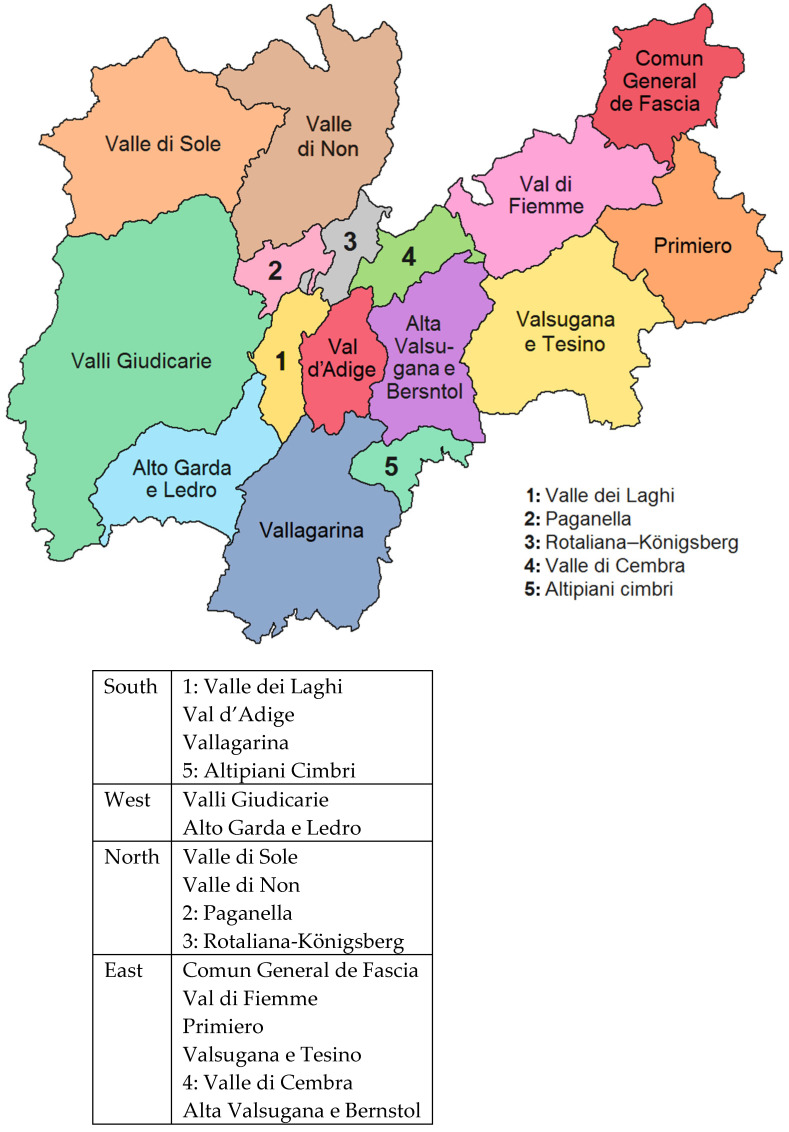
Geographical areas of the Province of Trento, Italy, and their aggregation according to South, West, North and East.

To identify contextual socio-environmental relationships, covariates were included, such as the population size of the communities where each nursing home was located [28]. In addition, since the area experiences a high influx of tourists, data on tourist arrivals for the 2019–2020 winter season were collected, specifically the number of customers, both Italians and foreigners, hosted in accommodation facilities (hotels or complementary), and the number of nights spent by customers in accommodation facilities, in the period from December 1, 2019 to February 29, 2020 for each municipality [28]. To examine the effect of regional differences in the geographic clustering of nursing homes on COVID-19 transmission, we included the geographic area of the province (North–South-East–West) in our analyses (see Figure 1).

### 2.4. Analyses

Descriptive statistics for continuous variables included the number of observations (nursing homes), the mean and standard deviation or median, and the first and third quartiles, when appropriate. For categorical variables, frequencies and percentages were presented. We compared the differences in characteristics between nursing homes that participated in the survey on IPC and those that did not participate. The size measure was converted from a continuous measure of number of beds to a categorical variable of ≤70 and >70 beds. We then compared the differences in characteristics between nursing homes that reported at least 1 confirmed COVID-19 case among residents and those that did not report any cases. We used the t-test for quantitative variables (i.e., total residents; total beds) and the Chi-square test, or the Fisher’s exact test when appropriate, for categorical variables (i.e., size; special care units). 

We then performed association analyses between the median cumulative incidence of COVID-19 cases among residents and the characteristics of the nursing homes that participated in the survey. We used the nonparametric Mann–Whitney U test for the comparison of two groups and the Kruskal–Wallis test for the comparison of more than two groups (i.e., geographical region). For the association analysis between quantitative variables (i.e., the population of the municipality) and the cumulative incidence of COVID-19 cases, we used the Spearman rho correlation coefficient; we used Pearson’s correlation coefficient for the association analysis of the cumulative incidence of COVID-19 between nursing homes and belonging municipalities. To evaluate the potential confounding of geographical area on the IPC measures, a two-level random intercept logistic model was fitted to the data, with level 1 units (patients in nursing homes) nested into level 2 units (nursing homes). The dependent variable was being a case of COVID-19 (dichotomous variable), and we included, as independent variables, the geographical area and the IPC measures separately in the model.

Analyses were conducted using Stata software, version 16 (StataCorp. 2019. Stata Statistical Software: Release 16. College Station, TX: StataCorp LLC). 

## 3. Results

The analysis included all 57 nursing homes in the province of Trento, comprising 5145 residents. The descriptive data of all nursing homes are depicted in Appendix A Table A1. Overall, 37 (64.9%) of the facilities had at least 1 resident COVID-19 case, with a median cumulative incidence of 4.8% (Interquartile Range—IQR: 0–40%). The majority of the facilities (63.2%) had more than 70 beds; 47.4% had special care units, of which 24.6% were dementia care units, and 22.8% were special units for residents with complex clinical conditions. More than a half of the facilities (56.1%) met the structural quality standards defined by the province. The majority of the homes were located in non-urban areas (68.4%) and predominantly in the southern region of the province (36.8%).

We also compared the main characteristics of nursing homes participating and not participating in the IPC survey (see Methods). The median cumulative incidence of outbreaks of COVID-19 in nursing homes was 4.8% overall; in nursing homes participating in the survey was 2.9%, and 17% among homes not participating, but this difference was not statistically significant. In general, the characteristics of homes participating and not participating in the IPC survey were similar, however facilities participating in the survey were more likely to be located in a rural area (*p* = 0.025).

Table 1 and Appendix A Table A2 report the information (including type of rooms, staffing, etc.) retrieved from the 45 nursing homes participating in the survey. With regard to IPC measures, the survey revealed suboptimal compliance in particular for specific training on infection control and prevention (24.4%), the presence of a committee for infection control and prevention (6.7%), and official documents on outbreak management (11.1%).

Table 2 describes the association between the median cumulative incidence of COVID-19 cases among residents and the characteristics of the nursing homes that participated in the survey. The median cumulative incidence for the nursing homes was not significantly associated with any of the considered variables, except for the geographical region (*p* = 0.002) where the homes were located: we found that the western region of the province showed the highest median cumulative incidence (45.2%).

The population size of the municipality, as well as the number of tourists and the number of nights spent by tourists in accommodation facilities where the nursing homes were located were not statistically associated to the median cumulative incidence of COVID-19 in nursing homes; this was observed also when correlating the number of staff units or the number of rooms to the cumulative incidence (Spearman’s rho from 0.038 to 0.240 in absolute values; data not shown). Moreover, the estimated Pearson correlation coefficient between the cumulative incidence of COVID-19 in municipalities and in nursing homes was 0.51 (0.54 when considering only nursing homes that participated in the survey).

Table 3 summarizes the results of the bivariate analyses for nursing homes that reported COVID-19 cases and those that did not report any cases. The 37 facilities that reported at least one case of COVID-19 had a greater average number of residents (95.8) than the 20 that did not report any cases (80.0) and a higher average number of beds (96.7 vs. 84.2, respectively). However, these differences were not statistically significant. 

**Figure 2:**
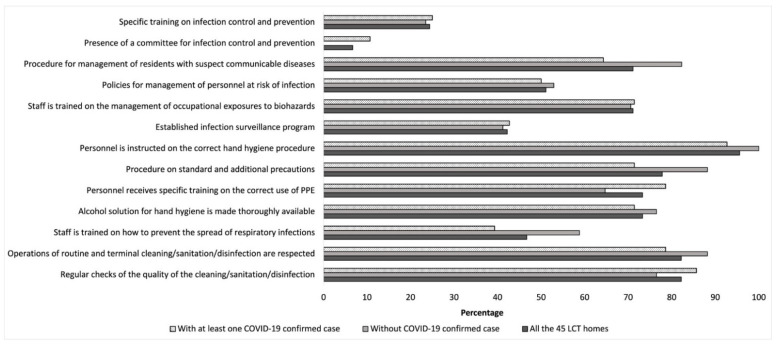
Percentage of LTC homes implementing infection control measures by presence of at least one COVID-19 confirmed case.

**Figure 3:**
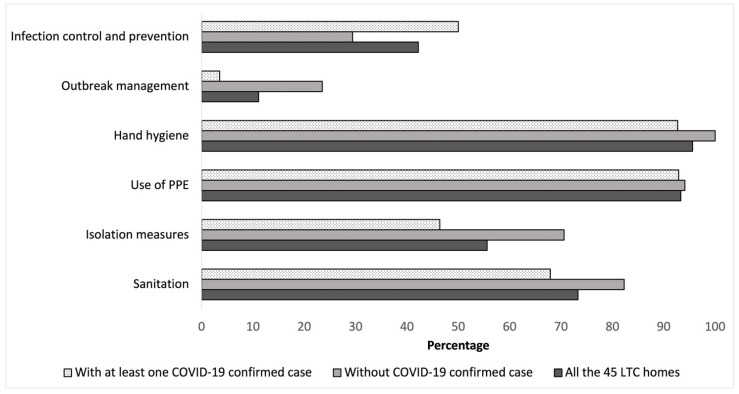
Percentage of LTC homes with official documents/guidelines by presence of at least one COVID-19 confirmed case.

Figure 2 and Figure 3 show the percentage of LTC homes by presence of infection control measures and guidelines/procedures. The nursing homes with no cases of COVID-19 were those who were more likely to implement outbreak management procedures (23.5%) compared to homes with at least 1 case of COVID-19 (3.6%) (*p* = 0.060).

The multivariate models indicated that none of the considered IPC measures was significantly associated to the likelihood of being a case of COVID-19 (Appendix A Table A3). In each model, the likelihood of being a case was significantly larger in the Western region with respect to the remaining areas of the province.

## 4. Discussion

The high COVID-19 morbidity and mortality observed among residents in nursing homes have posed a major challenge for specific strategies for disease prevention and control in such settings. Previous research showed that certain features and practices of nursing homes may affect care across a variety of outcome and process measures [29,30,31]. 

In our study, we cross-linked infection cases related to the first phase of the COVID-19 pandemic to evaluate how multiple “baseline” factors at the facility level and COVID-19 transmission in the local community may have been associated to the spread of COVID-19 infection in nursing homes located in the Autonomous Province of Trento. We performed the evaluation in a timeframe covering the entire “first wave” of the COVID-19 epidemic, when containment measures were not fully in place. Italy (and particularly Northern Italy) was hit hard and fast and the country ranked among those with the highest COVID-19 burden [22,23].

The cumulative incidence of COVID-19 was higher in the nursing home facilities located in the western area of the province, which borders the Lombardy region, the most affected area during the first phase of the pandemic [17,22]. We found an association between the cumulative incidence of COVID-19 in the general population and in nursing homes. 

The finding of a positive correlation between the cumulative incidence of COVID-19 in the surrounding community and outbreaks in long-term care facilities is in line with results reported in other studies [32,33,34,35]. 

In our study, no structural or organizational characteristics, including staffing levels, nor any IPC measure considered (i.e., training on IPC, established committee and/or infection surveillance programs, documents/guidelines on IPC and outbreak management) were significantly associated to an increase or decrease in the prevalence of COVID-19 in nursing homes. However, it should be noted that the nursing homes with no cases of COVID-19 were those who were more likely to have implemented outbreak management procedures (23.5%) compared to nursing homes with at least 1 case of COVID-19 (3.6%) (*p* = 0.060).

These results differ from those of other studies that identified structural and organizational factors related to an increased risk of COVID-19 infection among residents. Some of these factors, including location, larger facility size, nursing staff levels under the recommended standard, for-profit status, low overall quality rating, and movement of staff between facilities have been confirmed as detrimental in various publications [36,37,38,39,40,41,42,43,44,45]. 

Multiple studies were focused on the association between implementation of preventive measures and COVID-19 outbreaks, with contrasting results ranging from no association [46,47] to a notable association [48,49,50,51]. Regarding the association of COVID-19 infection cases with IPC measures and policies, we found no differences between the facilities that participated in the survey and those that did not participate. The only difference was that most of the participant facilities belonged to a rural rather than to an urban context; however, no significant differences related to this aspect were found in the cumulative incidences of COVID-19 cases. 

In our study, we collected data reflecting the actual situation and the degree of implementation of some IPC measures within the facilities showed sub-optimal levels of compliance. Our results are consistent with other studies carried out in a pre-pandemic context showing the need to tailor and enhance policies and practices related to IPC measures in nursing homes [52,53,54,55,56,57]. It is clear that COVID-19 has highlighted the need for quality improvements in these care settings and the need for the adoption of medical standards by several countries. Implementing comprehensive IPC programs and following critical care IPC strategies and precautions are crucial steps to prevent/limit SARS-CoV-2 transmission in health facilities. Standard precautions recommended as part of routine health care provided to all patients in all health care settings include hand hygiene, respiratory hygiene, appropriate use of PPI according to risk assessment, injection safety, decontamination and re-processing of medical equipment, environmental cleaning, and safe waste management [58]. Additional precautions recommended in the context of COVID-19 pandemic included maintaining a physical distance among all individuals (at least 1 mt.), transmission-based precautions (universal and targeted continuous masking), isolation and cohorting of patients with suspected or confirmed COVID-19, contact and droplet precautions (and airborne precautions according to risk assessment), administrative controls (i.e., prevention, identification and management of COVID-19 among healthcare workers, and management of visitors entry), and environmental and engineering controls (i.e., adequate indoor air quality, special separation) [58]. Optimal adherence to current IPC guidance is essential also in the context of current SARS-CoV-2 variants of concern, based on the available evidence and expert consensus.

It is worth noting that no element of the IPC measures assessed was associated to the median cumulative incidence of COVID-19 cases. We could explain our findings in the context of the extraordinary impact of the COVID-19 pandemic in Italy, with a sustained viral circulation across all of Northern Italy, including the Autonomous Province of Trento, as well as a very rapid spread of cases and deaths in the first phase (February–May 2020) in nursing homes [18]. In view of this pandemic, it could be hypothesized that no structural, staffing, or other identifiable factors related to ongoing prevention and control programs could have been crucial in effectively contrasting the spread of COVID-19 in nursing homes in Italy in the early stages of the pandemic. Moreover, Italian nursing homes during the “first wave” of the pandemic had to cope with multiple operational hurdles and management difficulties (i.e., a lack of personal protective equipment, an inability to have swabs tested, a lack of healthcare staff, difficulties in isolating COVID-19-infected residents, etc.), limiting the effective and timely application of measures that may have helped in containing the spread of the virus in these settings [18,59].

The lack of an association between IPC measures and the reporting of COVID-19 cases could also be explained by the exceptionality of the infection control measures needed. Moreover, outbreaks of newly emerging infectious diseases are a challenge and a threat to healthcare providers and other frontline responders due to both a limited understanding of the emerging threat and to a reliance on IPC measures that may not consider all of the transmission dynamics of the emerging pathogen [60]. 

It is essential to study the impact of COVID-19 on nursing homes to generate substantial knowledge and for its subsequent translation into policies and resource allocation decisions. Further studies are also needed to understand the spread of COVID-19 infection during different phases and “pandemic waves,” and in different contexts and long-term care settings. The accumulating evidence on COVID-19 in nursing homes needs to be leveraged in several ways to support public health and safety measures to protect the vulnerable populations who reside within these facilities against the risk of an epidemic during the current and future pandemics. A robust approach involves adequate staffing levels and competences, facility design, enhanced IPC capacity within nursing homes, as well as strengthening the public health surveillance system in the community to rapidly identify, assess and control epidemic outbreaks. These interventions should be part of a comprehensive and holistic model that balances infection control and quality of care with the well-being of residents and quality of life in residential care settings [61,62,63]. 

### Limitations

The collected data are related only to nursing homes in an Italian region during the initial phase of the COVID-19 pandemic. Information on the individual characteristics of the residents of nursing homes was not available for this analysis; however, the aim of the present study was to assess the effect of non-clinical factors associated with COVID-19 cases, not to investigate other resident outcomes (i.e., symptoms, illness severity or death), which could have been affected by other risk factors, alongside the reliability of data collected during the initial emergency phase. We did not investigate the association between COVID-19 cases among staff members and residents due to the insufficient reliability of collected data for some facilities, as well as unsystematic testing of asymptomatic staff members during the first part of the pandemic wave. We believe that such analysis could have further reinforced the linkage between cases among NH residents and spread of the infection in the local community.

The study was based on a retrospective analysis. Moreover, the information on IPC practices, although collected through a specific survey, was retrieved before the pandemic and therefore might not accurately reflect the situation in March 2020. The situation, however, has unlikely changed during that period. In addition, it is worth noting that most of the variables analyzed were based on existing policies, procedures, protocols, education and training activities, operational tools and collected data, representing only a (reliable) proxy of the effectiveness of IPC programs and of the appropriate implementation of evidence-based recommendations and good practices. Therefore, we did not perform any clinical assessment relying on direct observance of healthcare staff behavior, understanding of professional competence, or patient-level audit of care processes. Finally, there might be additional confounding factors regarding regulation, organization and practices that may have not been considered in the analysis, which could potentially be related to COVID-19 cases in nursing homes. At the same time, the governance and model of care for the elderly in nursing homes are standardized in the Autonomous Province of Trento. However, organization and care models across the Italian territory are heterogeneous, so it is difficult to generalize the results to the whole country or elsewhere.

## 5. Conclusions

The COVID-19 outbreak in this region confirmed the vulnerability of nursing homes in the face of the COVID-19 pandemic. In this national cohort study of long-stay nursing home residents, the risk of SARS-CoV-2 was associated with the geographic area. However, in our study, there were no other factors associated with COVID-19 infections.

To target resources and better respond to future outbreaks, a comprehensive, data-driven and trans-national understanding of factors that may affect the epidemiology of COVID-19 pandemic (or other infectious outbreaks) is critical to encourage the creation of policies and practices that may improve our preparedness and responsiveness in similar settings. In order to deal effectively with comparable scenarios in the future, it is necessary to design and plan tailored measures that can be implemented as quickly as possible if necessary, which should regularly be updated in cooperation with local public health authorities. 

Given the consequences of epidemic outbreaks, collaboration between nursing homes, public health authorities and social and health actors in the local community can yield solutions that guarantee pandemic preparedness, alongside models of care oriented toward residents.

## Figures and Tables

**Table 1:** Information on the infection prevention and control practices of the nursing homes retrieved by means of the infection prevention and control survey (N = 45).

Variables	
Infection prevention and control measures (presence of), n (% facilities)	
Specific training on infection control and prevention	11 (24.4%)
Presence of a committee for infection control and prevention	3 (6.7%)
Procedure for management of residents with suspected communicable diseases	32 (71.1%)
Policies for management of personnel at risk of infection	23 (51.1%)
Training of staff on the management of occupational exposures to biohazards	32 (71.1%)
Established infection surveillance program	19 (42.2%)
Training of staff on the correct hand hygiene procedure	43 (95.6%)
Procedure on standard and additional precautions	35 (77.8%)
Training of staff on the correct use of personal protective equipment (PPE)	33 (73.3%)
Availability of hand hygiene supplies	33 (73.3%)
Training of staff on how to prevent the spread of respiratory infections	21 (46.7%)
Compliance with operations of routine and terminal cleaning/sanitation/disinfection	37 (82.2%)
Official protocols/procedures on infection control and prevention	19 (42.2%)
Outbreak management	5 (11.1%)
Hand hygiene	43 (95.6%)
Use of PPE	42 (93.3%)
Isolation measures	25 (55.6%)
Sanitation	33 (73.3%)

**Table 2:** Association analyses between median cumulative incidence of COVID-19 cases among residents and characteristics of the nursing homes that participated in the survey (N = 45).

Variable	Median Cumulative Incidence of COVID-19 Cases (%) [p25–p75]	*p*
Facility size		0.930
Small–medium (≤70 beds), (16)	1.8 [0–48]	
Large (>70 beds), (29)	7.4 [0–36]	
General characteristics		
Special care units		0.174
yes (22)	19 [0–40]	
no (23)	1.1 [0–39]	
Dementia		0.421
yes (13)	14 [0–45]	
no (32)	1.6 [0–37]	
Complex clinical problems		0.444
yes (9)	24 [0.53–36]	
no (36)	1.8 [0–40]	
Conformity to quality standards		0.990
yes (25)	1.6 [0–42]	
no (20)	10 [0–32]	
Metropolitan status		0.567
Urban (11)	0.53 [0–36]	
Rural (34)	3.8 [0–42]	
Geographical region		0.002
North (5)	1.1 [0–44.6]	
South (15)	0 [0–24.2]	
East (14)	0.8 [0–4.8]	
West (11)	45.2 [24–55.9]	
Infection control measures		
Specific training on infection control and prevention		0.388
yes (11)	14 [0–47.9]	
no (34)	1.8 [0–28.1]	
Presence of a committee for infection control and prevention		0.081
yes (3)	47.9 [14–54.8]	
no (42)	1.8 [0–35.9]	
Procedure for management of residents with suspected communicable diseases		0.871
yes (32)	4.4 [0–39]	
no (13)	1.9 [0.5–35.9]	
Policies for management of personnel at risk of infection		0.341
yes (23)	1.6 [0–28]	
no (22)	13.5 [0–44.6]	
Training of staff on the management of occupational exposures to biohazards		0.952
yes (32)	3.8 [0–39.4]	
no (13)	1.9 [0–35.9]	
Established infection surveillance program		0.749
yes (19)	2.9 [0–24.5]	
no (26)	3.3 [0–42]	
Training of staff on the correct hand hygiene procedure		0.553
yes (43)	2.9 [0–38.5]	
no (2)	23.1 [1.6–44.6]	
Procedure on standard and additional precautions		0.365
yes (35)	1.6 [0–40.3]	
no (10)	18.6 [1.9–38.5]	
Training of staff on the correct use of PPE		0.301
yes (33)	4.8 [0–40.3]	
no (12)	0.8 [0–30]	
Availability of hand hygiene supplies		0.571
yes (33)	1.6 [0–35.9]	
no (12)	8.8 [0–44.9]	
Training of staff on how to prevent the spread of respiratory infections		0.269
yes (21)	4.8 [0–24.5]	
no (24)	2.4 [0–44.9]	
Compliance with operations of routine and terminal cleaning/sanitation/disinfection		0.553
yes (37)	1.9 [0–38.5]	
no (8)	9.4 [0.8–40]	
Regular checks of the quality of the cleaning/sanitation/disinfection		0.918
yes (37)	2.9 [0–35.9]	
no (8)	14.1 [0–43.3]	
Official protocols/procedures on		
infection control and prevention		0.123
yes (19)	24 [0–45.2]	
no (26)	1.6 [0–24.2]	
Outbreak management		0.087
yes (5)	0 [0–0]	
no (40)	5.4 [0–40.3]	
Hand hygiene		0.915
yes (43)	2.9 [0–40.3]	
no (2)	7.8 [1.6–14]	
use of PPE		0.742
yes (42)	3.8 [0–40.3]	
no (3)	0.5 [0–38.5]	
Isolation measures		0.941
yes (25)	2.9 [0–40]	
no (20)	3.3 [0.3–24.2]	
Sanitation		0.408
yes (33)	1.6 [0–40.3]	
no (12)	9.4 [0.8–31.4]	

**Table 3:** Bivariate analyses for nursing homes in the province of Trento that reported COVID-19 cases and those that did not report any cases.

Variable	Confirmed COVID-19 Cases		*p*
	Yes (N = 37)	No (N = 20)	
Residents, mean ± SD	95.8 ± 47.8	80.0 ± 30.3	0.188
Beds, mean ± SD	96.7 ± 48.0	84.2 ± 33.0	0.306
Facility size			0.716
Small–medium (≤70 beds), n	13 (35.1%)	8 (40%)	
Large (>70 beds), n	24 (64.9%)	12 (60%)	
General characteristics			
Special care units			0.169
yes, n	20 (54%)	7 (35%)	
no, n	17 (45%)	13 (65%)	
Dementia			0.749
yes, n	10 (27%)	4 (20%)	
no, n	27 (73%)	16 (80%)	
Complex clinical problems			0.346
yes, n	10 (27%)	3 (15%)	
no, n	27 (73%)	17 (85%)	
Conformity to quality standards			0.492
yes, n	22 (59.5%)	10 (50%)	
no, n	15 (40.5%)	10 (50%)	
Metropolitan status			0.683
Urban, n	11 (29.7%)	7 (35%)	
Rural, n	26 (70.1%)	13 (65%)	
Geographical region			0.100
North, n	6 (16.2%)	2 (10%)	
South, n	12 (32.4%)	9 (45%)	
East, n	8 (21.6%)	8 (40%)	
West, n	11 (29.7%)	1 (5%)	
Single-occupancy rooms			
(% over total rooms per facility), median [p25–p75]	18 [13–34]	27 [12–32]	0.620
Full-time equivalent nurses, median [p25–p75]	9.8 [7.3–14]	9 [7–10]	0.391
Full-time equivalent physicians, median [p25–p75]	1 [0.7–1.4]	1 [1–1]	0.732
Full-time equivalent aid staff, median [p25–p75]	37 [30–64]	38 [28–52]	0.622

## Data Availability

The datasets used and analyzed during the current study are available from the corresponding author upon reasonable request.

## Appendix A

**Table A1:** Main characteristics of the nursing homes (NH) in the province of Trento, by participation in the infection prevention and control survey.

Variable	NH (N = 57)	Participation in the Survey		*p*
		Yes (N = 45)	No (N = 12)	
Total residents ^#^, n Mean ± SD	5145 90.3 ± 42.9	4158 92.4 ± 45.1	987 82.3 ± 33.7	0.399 *
Total beds ^#^, n Mean ± SD	5263 92.3 ± 43.5	4273 94.9 ± 45.7	990 82.5 ± 33.7	0.305 *
At least 1 resident COVID-19 case, n	37 (64.9%)	28 (75.7%)	9 (24.3%)	0.410
Incidence of COVID-19 (%), median [p25–p75] ^°^	4.8 [0–40]	2.9 [0–39]	17 [1.2–46]	0.605
Facility size				0.697
Small–medium (≤70 beds), n	21 (36.8%)	16 (35.6%)	5 (41.7%)	
Large (>70 beds), n	36 (63.2%)	29 (64.4%)	7 (58.3%)	
General characteristics				
NH with special care units, n	27 (47.4%)	22 (48.9%)	5 (41.7%)	0.656
NH with dementia care units, n	14 (24.6%)	13 (28.9%)	1 (8.3%)	0.142
NH with care units for complex clinical problems, n	13 (22.8%)	9 (20%)	4 (33.3%)	0.328
Conformity to structural quality standards (yes), n	32 (56.1%)	25 (55.6%)	7 (58.3%)	0.863
Metropolitan status				**0.025**
Urban, n	18 (31.6%)	11 (24.4%)	7 (58.3%)	
Rural, n	39 (68.4%)	34 (75.6%)	5 (41.7%)	
Geographical region				0.274
North, n	8 (14%)	5 (11.1%)	3 (25%)	
South, n	21 (36.8%)	15 (33.3%)	6 (50%)	
East, n	16 (28.1%)	14 (31.1%)	2 (16.7%)	
West, n	12 (21.1%)	11 (24.4%)	1 (8.3%)	

* The *p*-value is related to the comparison of means. ^#^ Total number of residents and beds as of 1 March 2020. ° Cumulative incidence of confirmed COVID-19 cases among residents for the period from 1 March 2020, to 1 June 2020. Significant differences are marked in bold.

**Table A2:** Additional information on characteristics of the nursing homes retrieved by means of the infection prevention and control survey (N = 45).

Variables	
Total rooms, n (median) (IQR)	2372 (44) (38–61)
Single occupancy	581 (10) (4–19)
Double occupancy	1541 (31) (22–38)
Triple occupancy	125 (0) (0–3)
Quadruple and more occupancy	125 (0) (0–0)
Staff (units), median [p25–p75]	
Full-time equivalent nurses	9 [7–13]
Full-time equivalent physicians	1 [0.77–1]
Full-time equivalent nurse aides	38 [29–55]

**Table A3:** Odds Ratio with 95% CIs for the association between being a case of COVID-19 (dependent variable) with measures of infection prevention and control, adjusted for geographical area.

	OR (95% CI)
Specific training on infection control and prevention	1.9 (0.4; 10.4)
Presence of a committee for infection control and prevention	17.4 (0.9; 307.2)
Procedure for management of residents with suspected communicable diseases	0.8 (0.2; 4.0)
Policies for management of personnel at risk of infection	0.7 (0.2; 2.8)
Staff is trained on the management of occupational exposures to biohazards	0.6 (0.1; 3.2)
Established infection surveillance program	0.7 (0.2; 2.9)
Personnel are instructed on the correct hand hygiene procedure	0.1 (0.0; 4.0)
Procedure on standard and additional precautions	0.7 (0.1; 4.1)
Personnel receive specific training on the correct use of PPE	1.8 (0.3; 9.7)
Alcohol solution for hand hygiene is made thoroughly available	0.7 (0.1; 3.7)
Staff are trained on how to prevent the spread of respiratory infections	1.1 (0.3; 5.0)
Operations of routine and terminal cleaning/sanitation/disinfection are respected	1.2 (0.2; 8.1)
Regular checks of the quality of the cleaning/sanitation/disinfection	1.2 (0.2; 8.5)
Official documents on infection control and prevention measures	1.8 (0.4; 8.3)
Official documents on outbreak management	0.2 (0.0; 3.3)
Official documents on hand hygiene	0.2 (0.0; 5.9)
Official documents on use of PPE	5.7 (0.3; 100.9)
Official documents on isolation measures	1.3 (0.3; 6.2)
Official documents on sanitation	1.3 (0.2; 7.2)
