# Correction: Cazzoletti et al. Risk Factors Associated with Nursing Home COVID-19 Outbreaks: A Retrospective Cohort Study. *Int. J. Environ. Res. Public Health* 2021, *18*, 8434

**DOI:** 10.3390/ijerph182413175

**Published:** 2021-12-14

**Authors:** Lucia Cazzoletti, Maria Elisabetta Zanolin, Ilaria Tocco Tussardi, Mulubirhan Assefa Alemayohu, Ernesto Zanetel, Donatella Visentin, Luca Fabbri, Massimo Giordani, Giancarlo Ruscitti, Pier Paolo Benetollo, Stefano Tardivo, Emanuele Torri

**Affiliations:** 1 Department of Diagnostics and Public Health, University of Verona, 37134 Verona, Italy; lucia.cazzoletti@univr.it (L.C.); elisabetta.zanolin@univr.it (M.E.Z.); ilaria.toccotussardi@univr.it (I.T.T.); mulubirhanassefa.alemayohu@univr.it (M.A.A.); zanetelernesto@libero.it (E.Z.); donatellavisentin77@gmail.com (D.V.); 2 Azienda Provinciale per i Servizi Sanitari, Autonomous Province of Trento, 38123 Trento, Italy; luca.fabbri@apss.tn.it (L.F.); direzionesanitaria@apss.tn.it (P.P.B.); 3 Unione Provinciale Istituzioni per l’Assistenza, Autonomous Province of Trento, 38122 Trento, Italy; direttore@upipa.tn.it; 4 Department of Health and Social Policies, Autonomous Province of Trento, 38123 Trento, Italy; giancarlo.ruscitti@provincia.tn.it (G.R.); emanuele.torri@provincia.tn.it (E.T.)

The authors would like to make the following corrections to this paper [1].

Regarding the median cumulative incidence of COVID-19 among residents of the nursing homes located in the western region of the province, we made a typographical error in the original article, writing 45% instead of 45.2%. Corrections have been made to the Abstract and Results sections.

In the original article, there was a mistake in Table 2 as published. The median cumulative incidence of COVID-19 cases (%) [p25–p75] according to the geographical region where the nursing homes were located and according to the different infection control measures, was erroneously reported. The corrected Table 2 appears below. 

In the original article, there was a mistake in Table 3 as published. The percentages of nursing homes were not assigned to the correct geographical regions in the two groups of nursing homes with and without confirmed COVID-19 cases. The corrected Table 3 appears below. 

Finally, in the original article there was a mistake in Appendix A Table A1 as published. The percentages of nursing homes were not assigned to the correct geographical regions. The corrected Appendix A Table A1 appears below. 

**Table A1:** Main characteristics of the nursing homes (NH) in the province of Trento, by participation in the infection prevention and control survey.

Variable	NH (N = 57)	Participation in the Survey		*p*
		Yes (N = 45)	No (N = 12)	
Total residents ^#^, n Mean ± SD	5145 90.3 ± 42.9	4158 92.4 ± 45.1	987 82.3 ± 33.7	0.399 *
Total beds ^#^, n Mean ± SD	5263 92.3 ± 43.5	4273 94.9 ± 45.7	990 82.5 ± 33.7	0.305 *
At least 1 resident COVID-19 case, n	37 (64.9%)	28 (75.7%)	9 (24.3%)	0.410
Incidence of COVID-19 (%), median [p25–p75] ^°^	4.8 [0–40]	2.9 [0–39]	17 [1.2–46]	0.605
Facility size				0.697
Small–medium (≤70 beds), n	21 (36.8%)	16 (35.6%)	5 (41.7%)	
Large (>70 beds), n	36 (63.2%)	29 (64.4%)	7 (58.3%)	
General characteristics				
NH with special care units, n	27 (47.4%)	22 (48.9%)	5 (41.7%)	0.656
NH with dementia care units, n	14 (24.6%)	13 (28.9%)	1 (8.3%)	0.142
NH with care units for complex clinical problems, n	13 (22.8%)	9 (20%)	4 (33.3%)	0.328
Conformity to structural quality standards (yes), n	32 (56.1%)	25 (55.6%)	7 (58.3%)	0.863
Metropolitan status				**0.025**
Urban, n	18 (31.6%)	11 (24.4%)	7 (58.3%)	
Rural, n	39 (68.4%)	34 (75.6%)	5 (41.7%)	
Geographical region				0.274
North, n	8 (14%)	5 (11.1%)	3 (25%)	
South, n	21 (36.8%)	15 (33.3%)	6 (50%)	
East, n	16 (28.1%)	14 (31.1%)	2 (16.7%)	
West, n	12 (21.1%)	11 (24.4%)	1 (8.3%)	

* The *p*-value is related to the comparison of means. ^#^ Total number of residents and beds as of 1 March 2020. ° Cumulative incidence of confirmed COVID-19 cases among residents for the period from 1 March 2020, to 1 June 2020. Significant differences are marked in bold.

The authors state that apart from the indicated issues, the values reported in the original article are correct. The authors apologize for any inconvenience caused and state that the scientific conclusions are unaffected. The original article has been updated.

## Figures and Tables

**Table 2:** Association analyses between median cumulative incidence of COVID-19 cases among residents and characteristics of the nursing homes that participated in the survey (N = 45).

Variable	Median Cumulative Incidence of COVID-19 Cases (%) [p25–p75]	*p*
Facility size		0.930
Small–medium (≤70 beds), (16)	1.8 [0–48]	
Large (>70 beds), (29)	7.4 [0–36]	
General characteristics		
Special care units		0.174
yes (22)	19 [0–40]	
no (23)	1.1 [0–39]	
Dementia		0.421
yes (13)	14 [0–45]	
no (32)	1.6 [0–37]	
Complex clinical problems		0.444
yes (9)	24 [0.53–36]	
no (36)	1.8 [0–40]	
Conformity to quality standards		0.990
yes (25)	1.6 [0–42]	
no (20)	10 [0–32]	
Metropolitan status		0.567
Urban (11)	0.53 [0–36]	
Rural (34)	3.8 [0–42]	
Geographical region		0.002
North (5)	1.1 [0–44.6]	
South (15)	0 [0–24.2]	
East (14)	0.8 [0–4.8]	
West (11)	45.2 [24–55.9]	
Infection control measures		
Specific training on infection control and prevention		0.388
yes (11)	14 [0–47.9]	
no (34)	1.8 [0–28.1]	
Presence of a committee for infection control and prevention		0.081
yes (3)	47.9 [14–54.8]	
no (42)	1.8 [0–35.9]	
Procedure for management of residents with suspected communicable diseases		0.871
yes (32)	4.4 [0–39]	
no (13)	1.9 [0.5–35.9]	
Policies for management of personnel at risk of infection		0.341
yes (23)	1.6 [0–28]	
no (22)	13.5 [0–44.6]	
Training of staff on the management of occupational exposures to biohazards		0.952
yes (32)	3.8 [0–39.4]	
no (13)	1.9 [0–35.9]	
Established infection surveillance program		0.749
yes (19)	2.9 [0–24.5]	
no (26)	3.3 [0–42]	
Training of staff on the correct hand hygiene procedure		0.553
yes (43)	2.9 [0–38.5]	
no (2)	23.1 [1.6–44.6]	
Procedure on standard and additional precautions		0.365
yes (35)	1.6 [0–40.3]	
no (10)	18.6 [1.9–38.5]	
Training of staff on the correct use of PPE		0.301
yes (33)	4.8 [0–40.3]	
no (12)	0.8 [0–30]	
Availability of hand hygiene supplies		0.571
yes (33)	1.6 [0–35.9]	
no (12)	8.8 [0–44.9]	
Training of staff on how to prevent the spread of respiratory infections		0.269
yes (21)	4.8 [0–24.5]	
no (24)	2.4 [0–44.9]	
Compliance with operations of routine and terminal cleaning/sanitation/disinfection		0.553
yes (37)	1.9 [0–38.5]	
no (8)	9.4 [0.8–40]	
Regular checks of the quality of the cleaning/sanitation/disinfection		0.918
yes (37)	2.9 [0–35.9]	
no (8)	14.1 [0–43.3]	
Official protocols/procedures on		
infection control and prevention		0.123
yes (19)	24 [0–45.2]	
no (26)	1.6 [0–24.2]	
Outbreak management		0.087
yes (5)	0 [0–0]	
no (40)	5.4 [0–40.3]	
Hand hygiene		0.915
yes (43)	2.9 [0–40.3]	
no (2)	7.8 [1.6–14]	
use of PPE		0.742
yes (42)	3.8 [0–40.3]	
no (3)	0.5 [0–38.5]	
Isolation measures		0.941
yes (25)	2.9 [0–40]	
no (20)	3.3 [0.3–24.2]	
Sanitation		0.408
yes (33)	1.6 [0–40.3]	
no (12)	9.4 [0.8–31.4]	

**Table 3:** Bivariate analyses for nursing homes in the province of Trento that reported COVID-19 cases and those that did not report any cases.

Variable	Confirmed COVID-19 Cases		*p*
	Yes (N = 37)	No (N = 20)	
Residents, mean ± SD	95.8 ± 47.8	80.0 ± 30.3	0.188
Beds, mean ± SD	96.7 ± 48.0	84.2 ± 33.0	0.306
Facility size			0.716
Small–medium (≤70 beds), n	13 (35.1%)	8 (40%)	
Large (>70 beds), n	24 (64.9%)	12 (60%)	
General characteristics			
Special care units			0.169
yes, n	20 (54%)	7 (35%)	
no, n	17 (45%)	13 (65%)	
Dementia			0.749
yes, n	10 (27%)	4 (20%)	
no, n	27 (73%)	16 (80%)	
Complex clinical problems			0.346
yes, n	10 (27%)	3 (15%)	
no, n	27 (73%)	17 (85%)	
Conformity to quality standards			0.492
yes, n	22 (59.5%)	10 (50%)	
no, n	15 (40.5%)	10 (50%)	
Metropolitan status			0.683
Urban, n	11 (29.7%)	7 (35%)	
Rural, n	26 (70.1%)	13 (65%)	
Geographical region			0.100
North, n	6 (16.2%)	2 (10%)	
South, n	12 (32.4%)	9 (45%)	
East, n	8 (21.6%)	8 (40%)	
West, n	11 (29.7%)	1 (5%)	
Single-occupancy rooms			
(% over total rooms per facility), median [p25–p75]	18 [13–34]	27 [12–32]	0.620
Full-time equivalent nurses, median [p25–p75]	9.8 [7.3–14]	9 [7–10]	0.391
Full-time equivalent physicians, median [p25–p75]	1 [0.7–1.4]	1 [1–1]	0.732
Full-time equivalent aid staff, median [p25–p75]	37 [30–64]	38 [28–52]	0.622
